# Perforation of the Right Ventricle Induced by Pulmonary Artery Catheter at Induction of Anesthesia for the Surgery for Liver Transplantation: A Case Report and Reviewed of Literature

**DOI:** 10.1155/2009/650982

**Published:** 2009-12-31

**Authors:** Maria Auxiliadora-Martins, Erick Apinagés dos Santos, Daniel Adans Wenzinger, Gil Cezar Alkmim-Teixeira, Gerardo Cristino de M. Neto, Ajith Kumar Sankarankutty, Orlando de Castro e Silva, Olindo Assis Martins-Filho, Anibal Basile-Filho

**Affiliations:** ^1^Divisão de Terapia Intensiva, Departamento de Cirurgia e Anatomia, Hospital das Clínicas da Faculdade de Medicina de Ribeirão Preto, Universidade de São Paulo, 2o andar. Av. Bandeirantes, 3900 Bairro Monte Alegre, Ribeirão Preto, SP, Brazil; ^2^Unidade de Transplante Hepático da Divisão de Cirurgia Digestiva, Departamento de Cirurgia e Anatomia, Hospital das Clínicas da Faculdade de Medicina de Ribeirão Preto, Universidade de São Paulo, Ribeirão Preto, SP, Brazil; ^3^Laboratório de Biomarcadores de Diagnóstico e Monitoração, Instituto René Rachou, Fundação Oswaldo Cruz, Belo Horizonte, MG, Brazil

## Abstract

We report a case of a 45-year-old male patient diagnosed with liver cirrhosis by hepatitis C and alcohol, with a Child-Pugh score C and a model for end-stage liver disease (MELD) score of 27, and submitted to liver transplantation. The subject underwent insertion of the pulmonary artery catheter (PAC) in the right internal jugular vein, with technical difficulty concerning catheter advance. There was sudden hypotension, increase in central venous pressure (CVP), and decrease in SvO_2_ 15 minutes after the PAC had been inserted, followed by cardiorespiratory arrest in pulseless electrical activity (PEA), which was promptly assisted with resuscitation. Pericardiocentesis was performed without success, so the individual was subjected to a subxiphoid pericardial window, which led to output of large amounts of blood as well as PEA reversal to sinus rhythm. Sternotomy was performed; rupture of the apex of the right ventricle (RV) was detected, and suture of the site was accomplished. After hemodynamic stabilization, the patient was transferred to the ICU, where he developed septic shock and, despite adequate therapy, died on the eighteenth day after ICU admission.

## 1. Introduction

The pulmonary artery catheter (PAC), also known as Swan-Ganz catheter, was introduced into medical practice in 1970 [1]. The PAC can be inserted in patients within minutes and provides intensivists with extremely useful information about hemodynamic variables. Invasive hemodynamic monitoring is an important therapeutic guideline for major surgeries such as liver transplantation, even though it is not without risk. Complications can be related to the introduction of the catheter (arterial puncture, pneumothorax, hemothorax, and thoracic duct lesion) or the PAC itself (valve lesion, catheter knotting, rupture of the pulmonary artery, perforation of the right ventricle, and cardiac arrhythmias). Aggravations can also be related to PAC maintenance, such as infection, pulmonary artery aneurysm or rupture, thromboembolism, cardiorespiratory arrest, and right ventricle perforation [2]. Myocardial perforation, in turn, is a rare but potentially life-threatening complication [3, 4]. The most commonly reported complications related to the use of PAC include cardiac arrhythmia [5] pneumothorax [6], pulmonary embolism [7], infection [8], and pulmonary artery perforation [9–12]. Right ventricle perforation by PAC is rare [13] but potentially fatal. In heart surgery, the surgeon usually detects perforation during handling of the heart. Therefore, the exact time and cause of the lesion are difficult to determine [14]. 

This work reports a case of perforation of the apex of the right ventricle (RV) during PAC insertion prior to a liver transplantation procedure followed by fatal severe septic shock. 

## 2. Case Report

 A 45-year-old male patient diagnosed with liver cirrhosis by hepatitis C and alcohol, with a five-year history of tabagism and digestive hemorrhage, was admitted to hospital and evaluated for liver transplantation. Upon admission, the individual presented regular general state, a Child-Pugh score (C) and MELD score (27). The patient was conscious, oriented, eupneic, icteric, and afebrile. The patient denied abdominal pain; flapping was absent. Physical examination revealed weight (96 kg), blood pressure (120/80 mmHg), pulse rate (78 bpm), and respiratory rate (16 ipm). Cardiovascular and pulmonary auscultations revealed no alterations. Palpation showed flat abdomen with no pain; ascites was absent. The liver was palpable 3 cm below the right costal margin. The lower limbs presented slight edema. Pretransplantation laboratory tests upon admission revealed altered levels of serum albumin (2.7 g/dL), total bilirubin (17.26 mg/dL), direct bilirubin (13.05 mg/dL), alanina amino transferase (22 U/L), aspartate amino transferase (111 U/L), INR (1.83) and lactate (2.1 mmol/L), low platelet counts (40,000/mm^3^) along with normal renal function and electrolytes levels. Liver transplantation was indicated at the same day. As a routine procedure during liver transplantation, the PAC (model 774HF75; Edwards Lifesciences, Irvine, CA, USA) was inserted after a single puncture of the right internal jugular vein, with technical difficulties concerning visualization of the RV curve and catheter advance. 15 after minutes the PAC had been inserted, the individual presented sudden hypotension, increased CVP and decreased SvO_2_, followed by cardiorespiratory arrest in pulseless electrical activity (PEA), which was promptly assisted with resuscitation (cardiac massage, adrenalin, and atropine). Pericardiocentesis was performed without success. The cardiac surgery team subjected the patient to a subxiphoid pericardial window, which led to output of large amounts of blood and PEA reversal to sinus rhythm. Sternotomy was then performed; rupture of the right ventricle apex was detected, and suture of the site was accomplished. After hemodynamic stabilization, the individual was transferred to the ICU. Cardiorespiratory arrest, caused by cardiac tamponade due to RV lesion, lasted for about 20 minutes. 

Upon ICU admission, the subject was hemodynamically stable, no longer sedated, making use of vasoactive drugs. Physical examination revealed a pale (2+/4+), afebrile, icteric (3+/4+), Glasgow coma scale (3) patient with miotic, isocoric pupils responsive to stimulus with light. The individual was in anasarca and presented muscle spasms as well as muscle twitching suggestive of myoclonus of the Lance-Adams syndrome type (myoclonus by hypoxic encephalopathy). The subject was submitted to invasive mechanical ventilation. An electroencephalogram (EEG) was requested and demonstrated nonspecific diffuse encephalopathy, so phenytoin and clonazepam were prescribed. The electrocardiogram obtained upon hospital admission remained unaltered. In the ICU, the patient progressed with hemodynamic instability, oliguric acute renal insufficiency due to shock, and pulmonary edema. Laboratory tests demonstrated acute metabolic acidosis (pH = 7.23; PaO_2_ = 187 mmHg; PaCO_2_ = 41.3 mmHg; HCO_3_ = 16.8 mEq/L; Base excess = −9.6, SaO_2_ = 100%), hypocalcemia (ionic calcium = 0.97 mEq/L), and hyperlactatemia (lactate = 7.1 mmol/L).

On the following day, there was onset of fever as well as serohematic fluid flow through the mediastinal drain. Chest X-radiography revealed right basal consolidation. Bronchoscopy was performed for collection of bronchoalveolar lavage, which was negative. Therapy with teicoplanin 200 mg/day + cefepime 1 g/day was initiated. Hemoculture and urine culture were requested; both tested negative. Culture of the secretion extracted from the surgical site revealed growth of *Staphylococcus hemolytic*. 

On the 6th day after ICU admission the individual still presented low conscious state score and oliguria, with need for mechanical ventilation, so tracheostomy was performed. Brain computed tomography revealed bilateral ischemic lesions with loss of brain and cerebellar volume; chest tomography evidenced consolidation with signs of right inferior lobe volume loss probably due to inflammation or large-volume pleural effusion on the right. EEG was repeated and demonstrated severe diffuse disorganization of the basal activity with diffuse attenuation of the delta range predominance, consistent with postanoxic diffuse encephalopathy. After 18 days in the ICU, the patient maintained a Glasgow 3 score, hemodynamic instability, and anuria, which led to his death.

## 3. Discussion

Since the 1970s, the PAC has been the major choice for estimation of the cardiac output in intensive care therapy, although its utilization still remains highly controversial. Minor and major complications associated with PAC use have been reported to occur in 23% and 4.4% of insertions, respectively [5]. Among the fatal complications related to the PAC use, the rupture of the pulmonary artery (PA) is the most common report, with rare cases of myocardium perforation [3, 4].

In 1985, Robin [15] questioned the use of the Swan-Ganz catheter and, in 1987, this author proposed a moratorium on its widespread and indiscriminate utilization. Later, in 1996, Connors et al. [16] demonstrated an increase in mortality during the initial assistance delivered to critical patients.

Nowadays, PAC is widely employed in intensive care units to determine not only the cardiac output but also other equally important hemodynamic variables in critically ill subjects. The primary aim of this technique is to furnish information about the hemodynamic characteristics of shock states as well as provide guidelines for therapeutics. The Swan-Ganz catheter also provides other hemodynamic data such as pulmonary arterial pressure and cardiac function. However, indication for PAC must be carefully considered once it is an invasive procedure that naturally poses serious risks. During PAC insertion, care must be taken so as to inflate the balloon as soon as the atrial curve is noticed in the monitor, in order to avoid endocardial lesion in the tricuspid valve, as well as in the right atrium and ventricle. It has been suggested that the presence of a pulmonary artery pressure waveform and the ability to measure cardiac output and mixed venous oxygen saturation from the PAC do not exclude the possibility that it could still be perforating the right ventricle [17]. In our case, we noticed technical difficulties concerning visualization of the RV curve and therefore to monitor the catheter advance. The PAC was then over inserted in approximately 10 cm and caused the RV perforation. The patient did not present any evidences of valvar dysfunction or RV dilatation. Moreover, there was no evidence of manufacturing problems with the PAC. It is possible that the catheter balloon was not properly inflated and together with the PAC looping inside the RV may have caused the RV perforation. For all these reasons, we reenforce that the PAC should only be applied in cases when additional data that will change the medical conduct and eventually interfere with patient prognosis can be obtained. Less invasive techniques are not largely employed for various reasons, such as high cost, complexity, limited hemodynamic parameters, and inherent errors.

Alternative methods have been tested aiming at reducing the number of complications while maintaining reliable measurements. Among the various investigated methods is indirect calorimetry [18], which nowadays is one of the tools available for the early assessment of critically ill patients. However, it is still necessary that intensivists gain more experience of and disseminate this method, so that this bedside procedure can be more effectively used by a larger number of critical care physicians.

Other less invasive methods such as Vigileo/FloTrac, which estimates the cardiac output by analysis of the blood pressure wave, has already been investigated in individuals submitted to liver transplantation. According to Biais et al. [19], this technique does not correlate with PAC because it overestimates the cardiac output in subjects with low-vascular resistance, as in the case of liver transplantation patients, especially those with Child-Pugh scores B and C.

The use of less invasive approaches for the measurement of hemodynamic variables is proposed so that earlier access to data that can interfere positively in the prognosis of critically ill patients can be achieved, without added risks. This early and less invasive approach is crucial for rapid therapeutic interventions at the right time, in order to restore tissue perfusion before irreversible organic dysfunction takes place. 

Pulmonary artery catheterization with the use of Swan-Ganz catheters is an easy and rapid technique for bedside hemodynamic monitoring. However, its abuse has been associated with complications that can be avoided if it is used by experienced operators. The randomized clinical trials in patients with acute coronary syndrome, noncoronary highrisk patients (including noncardiac surgical patients and patients with sepsis and acute respiratory distress syndrome), and patients with chronic heart failure have established that its routine use is not necessary and may be associated with increased complications, including death. For heart and lung transplantation workup, hemodynamic monitoring is always necessary. In many institutions, hemodynamic studies are conducted before liver transplantation [20, 21].

## 4. Conclusion

The present case report is important for intensivists to reflect on the risks and benefits of invasive procedures carried out on critically ill patients. PAC remains as a reliable and widely employed method for the hemodynamic monitoring of individuals submitted to liver transplantation. Even though it is invasive, the Swan-Ganz catheter is extremely important for the hemodynamic management of such patients from the moment of anesthesia induction to arrival and stay in the ICU. Further studies comparing less invasive methods for hemodynamic monitoring shall be carried out, especially in cohorts of patients submitted to organ transplantation. 

##  Conflict of Interest Statement 

All authors have no conflict of interest to disclose. 
